# Rooting out bias

**DOI:** 10.7554/eLife.32014

**Published:** 2017-09-29

**Authors:** Bridget M Kuehn

**Affiliations:** eLifeChicagoUnited States

**Keywords:** peer review, bias, scientific publishing, funding agencies

## Abstract

Tackling unconscious bias is a major challenge for journals and the rest of the scientific community.

While many scientists pride themselves on not being biased, the data suggest otherwise. Study after study has found that women scientists and scientists from certain minorities experience bias when it comes to getting funded, getting published or getting on in their career. This bias can be both conscious and unconscious. And while many organizations have taken steps to eliminate conscious bias, even the most conscientious individuals are prone to unconscious bias. Within peer review and publishing bias can manifest itself by fewer women being selected as referees and fewer papers by women authors being cited by other papers.

Patricia M Knezek, chair of the committee on the status of women in astronomy at the American Astronomical Society, says that she has caught herself assuming that the author of a paper is male when only first initials are given. She attributes this to growing up in a culture that places less value on the contributions of women and minorities. "It’s subtle," she says, "but there is a tendency to intrinsically devalue women’s work." Knezek argues that many different stakeholders – individual scientists, employers, funding agencies, journals and learned societies – need to help by actively monitoring for signs of bias and taking steps to counteract it at the level of both individuals or organizations.

"Bias is bad for meritocracy in science," says Andreas Neef of the Max Planck Institute for Dynamics and Self-Organization in Göttingen. Neef was a co-author on a recent paper which found that women were underrepresented as editors, reviewers and authors, even when adjustments were made for the lower participation of women in science overall (Helmer et al., 2017). The study, which was conducted on a sample of 9,000 editors and 43,000 reviewers from the Frontiers series of journals, found that the situation was improving with time, albeit very slowly: based on current trends the underrepresentation of women could persist until 2027 for authors, 2034 for reviewers and 2042 for editors. "The situation is improving," says Neef, "but without interfering in the trend it will take too long to reach equity."

## Underrepresentation is widespread

Neef's study is one of many across a variety of disciplines to document the underrepresentation of women in peer-reviewed journals. An analysis of the 20 earth and space science journals published by the American Geophysical Union (AGU) between 2012 and 2016 found that 27% of first authors were women, and that 28% of AGU members were women: however, only 21% of reviewers were women (Lerback and Hanson, 2017). And a study of more than 200,000 papers published in five major astronomy journals between 1950 and 2015 found that women authors received 10% fewer citations (Caplar et al., 2017).

Although many of these studies were not designed to tease out the cause of this underrepresentation, they do point to potential clues. Neef and colleagues found that male and female editors had a tendency to select reviewers of the same gender: men were selected by male editors to review 73% of the time, and female editors selected women reviewers 33% of the time. The female-female preference was driven by a small number of female editors who chose women reviewers at a very high rate, says Neef.

Women scientists are also less likely to have their grants funded, as are scientists from certain minorities

A preference for like individuals is a common human trait, and is likely to extend to other areas like hiring, grant funding and promotions. "It’s a hard thing to fight because you are comfortable working with people who are like you," says Knezek. But if left unabated it could have detrimental effects on peer review and science more generally, by reducing the ability of women or other underrepresented groups to advance or stay in scientific careers.

Women scientists are also less likely to have their grants funded, as are scientists from certain minorities (Ginther et al., 2011). Patricia Devine of the University of Wisconsin-Madison and colleagues recently completed collecting data for a study on whether identical grant applications with names that are indicative of a white male, white female, black maleor black female will be graded differently by reviewers for the US National Institutes of Health (NIH). She hopes the study will be able to tease out whether the lower funding rates are caused by differences in quality or by bias, which will help the NIH develop interventions. "If scientists aren’t being treated equally because of their gender or race we will not be in a position to fund the very best scientists," says Devine. "That can threaten the integrity of the scientific literature."

Devine and her colleagues have also developed tools to help individuals and organizations identify and mitigate biases. These include exercises that help individuals recognize unconscious bias and help them to focus on an individual’s merits rather than their gender or race. To test this approach, they conducted a randomized trial at the University of Wisconsin that involved offering a workshop on the tools to faculty in some departments but not others (Carnes et al., 2015). After this intervention, faculty in departments who were offered the workshop were more likely to report taking action against bias than faculty in the control departments. "They are more tuned in and aware of their own proclivity for bias," says Devine. "When you are tuned in you can reach for a tool."

Both men and women faculty members in intervention departments reported greater satisfaction with their work environment. The hiring of women, which was about 32% in all departments prior to the trial, also increased by 15% in the intervention departments. "Men and women both felt that their work was more respected in intervention departments than control departments," says Devine. "They felt they could raise issues like family needs without being stigmatized." However, Devine stresses that this type of intervention can only address unconscious bias – it will not help to address overt bias, such as people who openly express the view that women are inferior in science.

## The way ahead

Making progress towards gender equality in peer review and science more generally will require a concerted effort on the part of journals, professional societies and individual scientists to root out bias. Neef suggests that all journals should conduct the type of analysis that he and his colleagues did, and recommends the use of pop-up reminders and other mechanisms to gently nudge the behavior of editors or reviewers. At the recent International Congress on Peer Review in Chicago Jory Lerback, formerly of the AGU, reported how asking authors to think of women and underrepresented groups when suggesting reviewers for their papers resulted in a statistically significant increase in the number of women reviewers suggested by male authors submitting to Geophysical Research Letters.

Professional organizations should also be looking at the representation of women and minorities as speakers, poster presenters and participants at scientific meetings, and setting up both formal and informal support networks for people from underrepresented groups at such meetings, says Knezek. And in addition to policing their own tendency for bias, she says it is important for scientists to speak up when they see bias, even if it is uncomfortable.

## Note

This Feature Article is part of a collection of articles on peer review.
